# Development of recombinant proteins for vaccine candidates against serotypes O and A of Foot-and-Mouth Disease virus in Bangladesh

**DOI:** 10.1099/acmi.0.000713.v4

**Published:** 2024-06-24

**Authors:** Salma Akter, M. Shaminur Rahman, M. Rafiul Islam, Masuda Akther, Humaira Anjume, Mafruha Marjia, Md. Mizanur Rahaman, M. Anwar Hossain, Munawar Sultana

**Affiliations:** 1 Department of Microbiology, University of Dhaka, Dhaka-1000, Bangladesh; 2 Department of Microbiology, Jahangirnagar University, Savar, Dhaka-1342, Bangladesh; 3 Department of Microbiology, Jashore University of Sciences and Technology, Jashore, Bangladesh; 4 Vice-Chancellor, Jashore University of Science and Technology, Jashore-7408, Bangladesh

**Keywords:** adaptable vaccine candidate, emerging lineages, foot-and-mouth disease virus, multiple epitopes, recombinant protein, serotype O and A

## Abstract

Frequent vaccine failure leading to recurrent outbreaks of Foot-and-Mouth Disease (FMD) in livestock populations necessitates the development of a customizable vaccine platform comprising potential antigenic determinants of circulating lineages of FMD viruses. Artificially designed, chimaeric protein-based recombinant vaccines are novel approaches to combat the phylogenetically diverse FMD Virus (FMDV) strains. Among seven recognized serotypes, only serotypes O and A are dominantly circulating in Bangladesh and neighbouring countries of Asia, where transboundary transmission, recurrent outbreaks and emergence of novel lineages of FMDV are highly prevalent. The objective of this study was to develop multi-epitope recombinant proteins, procuring immunogenicity against circulating diverse genotypes of FMDV serotypes O and A. Two chimaeric proteins, named B1 (41.0 kDa) and B3 (39.3 kDa), have been designed to incorporate potential B-cell and T-cell epitopes selected from multiple FMDV strains, including previously reported and newly emerged sub-lineages. After expression, characterization and immunization of guinea pigs with a considerable antigen load of B1 and B3 followed by serological assays revealed the significant protective immunogenicity, developed from the higher (100 µg) doses of both antigens, against most of the currently prevalent serotype O and A strains of FMDV. The efficient expression, antigenic stability, and multivalent immunogenic potency of the chimaeric proteins strongly indicate their credibility as novel vaccine candidates for existing serotypes O and A of FMDV in Bangladesh and surrounding territories.

## Data Summary

No new sequence data was generated or published during this study. The sequences and orientation of the artificial proteins B1 and B3 are provided in Fig S2 and S3, available with the online version of this article. Previously published sequences used to support the analysis of this manuscript are listed in the supplementary file and can be accessed from the NCBI GenBank database (www.ncbi.nlm.nih.gov).

## Introduction

Foot-and-Mouth Disease (FMD) is recognized as a highly transmissible disease that infects more than 70 species of cloven-hoofed animals and causes devastating economic loss in FMD-endemic countries worldwide [1]. Recent outbreaks in several countries like the United Kingdom, Japan, China and South Korea have again urged for developing effective mitigation strategies against this deadly disease [2].

FMD virus (FMDV) is a member of the *picornaviridae* family, possessing a positive-sense single-stranded RNA genome and ‘error-prone’ RNA polymerase which confers the characteristic high mutation rate and the emergence of new variants. Seven antigenically different serotypes of FMDV (serotypes O, A, C, Asia1, Southern African Territories [SAT] 1, 2 and 3) are circulating across the world with diverse genotypes such as topotypes, lineages, and sub-lineages [3]. Serotypes O, A, and Asia1 have a worldwide dominant distribution along with continuous evolution and phylogenetic differentiation [4]. Specifically, serotype O viruses are responsible for the majority of outbreaks globally, followed by serotype A. Thus, these two serotypes warrant special efforts to reduce the global burden of FMD [5,6].

Currently, inactivated viral vaccines are the most widely used strategy throughout the world to control FMD and achieve the goal of the Progressive Control Pathway for Foot-and-Mouth Disease (PCP-FMD) jointly proposed by FAO and OIE. However, several limitations of the inactivated whole virus vaccine pose potential threats to its acceptability. For instance, the possibility of virus escape during production, inactivation, storage and subsequent transport conditions impose potential hazards for inactivated virus vaccine manufacturers [7]. Additionally, removing non-structural proteins (NSPs) from purified inactivated antigen preparations is crucial for pre- and post-vaccination differentiation of infected and vaccinated animals. Moreover, the vaccine strain must be updated frequently to defend against the most recently emerged strains of FMDV in susceptible areas. The lack of vaccine strain upgradation, the emergence of phylogenetically distinct subgroups and their transboundary prevalence give rise to frequent vaccine failure incidences in different countries of the world [8]. Recently, the emergence and transboundary transmission of two novel sub-lineages Ind-2001BD1 (or Ind-2001e) and Ind-2001BD2 of serotype O [9], novel lineage BD-18 (G-IX) of Asia1 in Bangladesh [10] strongly implicates the necessity of effective FMD vaccine development with potential immunogenicity to combat the repetitive outbreaks of FMDV by diverse circulating strains of serotype O and serotype A.

For these reasons, many research groups worldwide are investigating scopes of developing alternative effective vaccine approaches for FMD, such as the development of multi-epitope recombinant subunit vaccines [11]. These epitope-based vaccines are emerging as promising candidates for future vaccines due to the absence of any risks of virus replication, the capability to function as DIVA vaccines, devoid of any residual proteins from whole viruses, and most importantly the combination of multiple epitopes on a single polyprotein platform [12]. Four structural proteins form the capsid of FMDV which are VP1, VP2, VP3 and VP4. Among them, VP1 comprises the crucial conformational B-cell epitopes for viral entry and subsequent lysis of infected host cells [13]. Notably, the G-H loop (amino acid [aa] positions 134–160), the B-C loop (aa position: 40–60) and the C-terminal (aa positions: 190–210 approximately) of VP1 is critical for eliciting neutralizing antibodies against FMDV in the infected hosts [14]. The highly conserved receptor binding site, the RGD (Arg-Gly-Asp) motif within the VP1 G-H loop plays a significant role in viral entry into host cells and inducing protective immunity in the host [15,17]. Several artificial vaccines based on epitopes from the G-H loop of VP1 of FMDV have shown neutralizing efficiency in guinea pigs, swine, etc. [18,20]. However, none of these endeavours incorporated the epitopes from novel lineages and sub-lineages that emerged in Bangladesh and spread to [21] many other countries.

Therefore, in this study, we have designed two complete artificial chimaeric proteins, B1 and B3, namely, composed of the surface epitope fragments, the G-H loop and the C-terminal of VP1 capsid proteins, of prevailing FMDV serotype O (B1) and A (B3) strains, representing different clusters of their phylogroups. The recombinant proteins possess conformational B-cell epitopes, like G-H loop and C-terminal, as well as two crucial T-cell epitopes that are assumed to enhance T-cell dependent immunity and cytotoxic T-cell proliferation. Also, two universal T cell epitopes, PADRE [21] and Invasin [22], have been included to further enhance T cell activity and memory cell development for long-term immunity [8,18]. The chimaeric proteins have been efficiently expressed in a prokaryotic system with significant yield and conformational stability. Guinea pig models have been immunized with vaccines formulated with the chimaeric proteins B1 and B3. Serological assays exhibited broad neutralizing efficacy of the vaccines of B1 and B3 against the viruses, including the newly emerged strains of serotypes O and A in recent years. This study represents the first comprehensive approach for vaccine candidate development using a novel combination of epitopes from circulating strains in Bangladesh, one of the most FMD-endemic countries in Asia.

## Methods

### Designing and *in silico* analysis of chimaeric proteins B1 and B3

#### Construction of chimaera B1 and B3

During this study, emphasis has been given to both predominant FMDV serotypes O and A, mainly transmitting in Bangladesh. A total of forty VP1 nucleotide sequences from serotype O and twelve from serotype A sequence data of FMDV (Table S1 and S2) were obtained from the National Centre for Biotechnology Information (NCBI) GenBank sequence database [23]. These sequences were obtained from FMDV strains isolated from Bangladesh previously by the Microbial Genetics and Bioinformatics lab (MGBL-109) of the Department of Microbiology, University of Dhaka (www.microbialgen.du.ac.bd). Several groups with different sequence homology in VP1 regions were revealed after performing multiple sequence alignment, protein variability analysis (Fig. S1) and phylogenetic relationship detection. From those groups, two types of fragments, G-H loop fragment (aa 128–164) and C-terminal fragment (aa 190–211) were selected as conformational B-cell epitopes to construct two multi-epitope recombinant proteins, namely B1 and B3. A total of six G-H loop fragments and three C-terminal fragments were oriented along with two T-cell epitopes, one from the N-terminal of VP1 and another from 3A proteins of FMDV strains (Tables1  2). In both B1 and B3 constructs, two universal T-cell epitopes, 12 amino acids long PADRE (Pan-human leucocyte Antigen DR-binding Epitope) and 16 amino acids long Invasin (from *Yersinia* spp.), were included at 5′ and 3′ of both chimaeras, respectively. The GEDG spacer sequence differentiated each epitope of B1 for proper spatial orientation of all immunogenic fragments. In B3, four spacer sequences were GEDC, and the remaining eight spacers were GEDG. Subsequently, the DNA sequences encoding both B1 and B3 were obtained by reverse genetics tools. Codon optimization is very crucial for the heterologous expression of eukaryotic pathogenic proteins through the bacterial system. B1 and B3 encoding DNA were optimized for codon usage using the OptimumGene algorithm (www.genscript.com) to ensure the influences of factors like codon usage bias, mRNA secondary structure, ribosomal binding sites, repeat sequences and restriction sites etc. Optimization of these parameters ensures a significant level of heterologous expression of both B1 and B3.

**Table 1.** Source of G-H loop, C-terminus of the VP1 proteins from circulating FMDV serotype O strains and universal T-cell epitopes

Epitopes	Proteins sequences	Origin of epitopes
PADRE	AKFVAAWTLKAAA	Universal T-cell epitopes [21]
VP1(128–164)	TVYNGN**C**KYGEGAVTNVRGDLQVLAQKATRTLPTSFN	G-H loop of BAN/TA/Dh-301/2016
NTTCE	VTPQNQINVL	N-terminal T cellepitopes of type O [13]
VP1(128–164)	TVYNGN**C**KYGEGAVTNVRGDLQVLAQKATRTLPTSFN	G-H loop of BAN/SI/Sh-234/2015
VP1(190–211)	PLLAIHPEQARHKQKIVAPVKQ	C-terminal of BAN/TA/Dh-301/2016
VP1(128–164)	TVYNGN**C**KYGESNVPNVRGDLQVLAQKAARPLPTSFN	G-H loop of BAN/TA/Ma-200/2014
VP1(190–211)	PLLAIHPSEARHKQKIVAPVKQ-	C-terminal of BAN/TA/Ma-200/2014
VP1(128–164)	TVYNGN**C**KYGEGAVTNVRGDLQVLAQKAARTLPTSFN	G-H loop of BAN/BO/Na-162/201
VP1(190–211)	PLLAIHPGQARHKQKIVAPVKQ	C-terminal of BAN/MG/Sa-287/2016
VP1(128–164)	TVYNGN**C**KYGEGAVTNVRGDLQVLAQKAARTLPTSFN	G-H loop of BAN/MG/Sa-287/2016
3A	AAIEFFEGMVHDSIK	Structural 3A proteinsT cells epitopes [13]
VP1(128–164)	TVYNGN**C**KYGGSDVANVRGDLQVLAQKAARPLPTSFN	G-H loop of BAN/PA/Ch-228/2015
Invasin	TAKSKKFPSYTATYQF	Universal T-cell epitopes [22]

NCBI accession no of FMDV strains are available in Supplementary File (Tables 1 and 2).

**Table 2.** Source of G-H loop, C-terminus of the VP1 proteins from circulating FMDV serotype A strains and universal T-cell epitopes

Epitopes	Sequences of epitope fragments	Origin of epitopes
VP1(129–165)	VYNGTNKYSAASGRARGDLGQLAARVAAQLPASFNFG	G-H loop of BAN/CH/Sa-304/2016
NTTCE	VKIGNVSPT	N-terminal T cell epitopes of Type-A [13]
VP1(129–165)	VYNGTNKYSAASGRVRGDLGQLAARVAAQLPASFNFG	G-H loop of BAN_CH_Ra-15_2012
VP1(192–211)	AVEVSSQDRHKQKIIAPAKQ	C-terminal of BAN/CH/Sa-304/2016
VP1(129–165)	VYNGTNKYSAASGRVRGDLGQLAARVAAQLPASFNFG	G-H loop of BAN_CH_Ra-16_2012
VP1(192–211)	AVEVSSQDRHKQKIIAPAKQ	C-terminal of BAN_CH_Ra-16_2012
VP1(129–165)	VYNGTNKYSAASGRVRGDLEQLAARVAAQLPASFNFG	G-H loop of BAN_CH_Ra-18_2012
VP1(192–211)	AVEVLSQDRHKQKIIAPAKQ	C-terminal of BAN_GA_Sa-197_2013
VP1(129–165)	VYNGTNKYSAASGRVRGDLGQLAARVAAQLPASFNFG	G-H loop of BAN_GA_Sa-197_2013
3A	AAIEFFEGMVHDSIK	3A of structural proteins [13]
VP1(129–165)	VYNGTNKYSAASGRVRGDLGQLAARVAAQLPASFNFG	G-H loop BAN_CH_Ra-08_2012

#### Prediction and analysis of the secondary and tertiary structures of B1 and B3

The Mfold web-based software (mfold.rna.albany.edu) was used to analyse the structures of folding messenger RNAs of the chimaeric genes [23], whereas the RNAfold (rna.tbi.univie.ac.at) and Genebee (www.genebee.msu.su/services/ rna2_reduced.html) online servers revealed the energetic stability of the predicted structures. Results were confirmed by CentroidFold web server, which indicated the mRNA was stable enough for effectual translation in the prokaryotic host [24]. GOR-IV (gor.bb.iastate.edu/) [25] and Jpred 4 (compbio.dundee.ac.UK/jpred/) [26] online servers were used for prediction of secondary structure of B1 and B3 proteins. In order to analyse the sequence and predict the protein structures and functions, the percentage of random coils, alpha-helices and beta-sheets, and the solvent accessibility were evaluated by the PredictProteins server [27].

The 3D structures of B1 and B3 were predicted based on amino acid sequences using the PONDEROSA-C/S, a server-based software package for automated proteins 3D structure determination. Results were examined by using Ponderosa Analyzer. The relevant results were viewed with PyMOL molecular graphics systems [28]. The 3D structures were evaluated using Ramachandran plot analysis [29]. Various physical and chemical parameters of B1 and B3 were computed (Table S4) using ExPASy - ProtParam tool [30].

### Synthesis, expression and characterization of recombinant chimaeric proteins B1 and B3

#### Transformation and expression of B1 and B3

The codon-optimized DNA constructs of B1 and B3 were then commercially synthesized from GenScript (www.genscript.com) with two restriction sites EcoR1 and Hindlll, respectively, at 5′ and 3′ end of both chimaeric proteins’ sequences. The artificial DNA fragments were inserted into the pET21a+ vector (Fig. S2) at the respective restriction site to produce recombinant plasmids pET21a+/B1 and pET21a+/B3. The pET-21a (+) vector carries an N-terminal T7-Tag sequence plus a C-terminal His-Tag sequence. The frameshifting was also checked before performing the cloning reaction. The freeze-dried recombinant plasmids pET21a+/B1 and pET21a+/B3 were reconstituted according to the manufacturer’s protocol. Both recombinant plasmids pET21a+/B1 and pET21a+/B3 were transformed into chemically competent DH5α cells for propagation. The plasmids were purified by PureYield Plasmid Miniprep System (Promega, USA). The molecular weight of the recombinant plasmid was detected by agarose gel electrophoresis. The purified plasmids were further transformed into chemically competent BL21(DE3) cells using the heat-shock process, and starter cultures were developed from transformed colonies isolated from ampicillin containing LB medium. Expression was induced in LB broth containing 1 % glucose when OD600 reached 0.4–0.6. Then 1 mM IPTG (Invitrogen, USA) was added for induction, and the 50 ml culture was incubated at 37 °C in a shaker at 150 r.p.m. for 4 h. No IPTG solution was added to the control sample but was also incubated with the test sample. After induction, all cultures were transferred to 50 ml falcons and cells were harvested by high-speed centrifugation. The harvested cells were then resuspended in phosphate buffered saline (PBS) and kept at −80 °C overnight.

#### Extraction, purification and quantification of chimaeric B1 and B3

The next day, the harvested cells were subjected to one freeze-thaw cycle followed by sonication (OmniRuptor, Germany; 100W, 10 s pulse, 5 min) for cell lysis and extraction of total proteins. After sonication, centrifugation was done to separate soluble and insoluble fractions at 14 000 r.p.m. for 15 min at 4 °C. The protein expression was first assessed through SDS-PAGE analysis of total extracts of all induced and control cells. According to the manufacturer’s protocol, each of the insoluble fractions, i.e. the pellets, were subjected to proteins purification under denaturing conditions by HisPur Ni-NTA Resin (Thermo Scientific, USA). The final elution was performed with 500 mM imidazole (Sigma, USA), and further desalting (ZebaSpin Desalting column, 7 KDa MWCO, Thermo Scientific, USA) the eluted fractions removed the imidazole and exchanged the buffer with PBS (pH 7.4; Gibco). The purified proteins were quantified by Bradford Assay (Thermo Scientific, USA) and stored at −80 °C until further use.

### Characterization of expressed B1 and B3 proteins

The molecular weights of purified B1 and B3 were determined by 12.5 % SDS-PAGE using the Mini-PROTEAN system (Biorad). The molecular weight of B1 and B3 were revealed to be 41.0 kDa and 39.3 KDa, respectively, which is very close to the predicted MW of both. The presence of a 6X-Histidine tag at the C-terminal of purified proteins B1 and B3 was also confirmed by Western blot techniques. Two micrograms of purified proteins were separated by SDS-PAGE using 12.5 % polyacrylamide gels. The separated proteins were transferred to a nitrocellulose membrane, 0.45 µm (BIO-RAD) using Trans-Blot SD Semi-Dry Transfer Cell (BIO-RAD). The membranes were incubated for 1 h with SuperBlock Blocking Buffer (Thermo Scientific, USA) at room temperature with moderate shaking. Then membranes were incubated with primary antibody, anti-6X-His tag mouse monoclonal antibody (Invitrogen, USA) at a dilution of 1 : 1000 for 1 h at room temperature in a mini-rocker. After washing the membrane five times (5 min each) with 0.1 % TBS-T, the membrane was incubated for 1.0 h at RT, with HRP-conjugated goat anti-mouse secondary antibody (Thermo Scientific, USA) (at a dilution of 1 : 10 000) directed against the primary antibody. Then the membrane was washed for five times using 0.1 % TBS-T to remove non-specifically bound secendary antibodies. SuperSignal West Pico PLUS Chemiluminescent Substrate (Thermo Scientific, USA) was used for detection according to the manufacturer’s protocol, and the final images were taken in ChemiDoc Imaging System (Biorad). Furthermore, the WB method has confirmed the presence of antigenic fragments on the purified proteins using serotype-specific monovalent anti-FMDV bovine serum as the primary antibody collected from repository of lab and anti-bovine goat antibody (HRP) (Thermo Scientific, USA) as the secondary antibody

### Immunogenicity testing of chimaeric B1 and B3

#### Experimental animals and viruses

For immunogenicity assessment of the recombinant proteins, a total of forty-nine Dunken-Hartley strains of guinea pig animal models (bodyweight 350–450 g) were used for immunization experiments. All experimental works were performed in the Animal Resource Facility of International Centre for Diarrheal Disease Research, Bangladesh (ICDDR’B). Animal handling for immunization and blood collection was performed according to the guidelines prescribed by the Ethical Review Committee of the University of Dhaka. Ethical clearance for the research works was obtained from the Ethical Review Committee, the University of Dhaka, approved by Animal Resource Facility (ARF), ICDDR’B.

For assessment of virus neutralization efficiency of the chimaeric proteins, two recently reported vaccine FMDV strains for both serotype O (BAN/TA/Dh-301/2016) and serotype A (BAN/CH/Sa-304/2016) have been collected from the repository of MGBL and revived using BHK-21 (ATCC no#BHK-21[C-13]-CCL-10) cell line [31,32]. The infectivity and tissue culture infective dose 50 (TCID_50_) were determined for each strain. Besides, four other strains, previously collected from field samples in Bangladesh, were also revived: BAN/NA/Ha-156/2013, BAN/BO/Na-161/2013, BAN/GA/Sa-197/2013 and BAN/DH/Sa-310/2017.

#### Antigen preparation

For the two recombinant proteins, four different concentrations of each antigen were inoculated in different groups of experimental guinea pigs. The doses were 100, 50, 10 and 2 µg per dose of antigen. The purified proteins were diluted in PBS (pH 7.4) and mixed with adjuvant Montanide201 in a 1 : 1 (w/w) ratio. The final proteins concentrations in the formulated antigen were obtained as 100, 50, 10 and 2 µg per 1.0 ml antigen dose. Eight antigen formulations were prepared, four of which were B1 and others were B3. The antigen preparations were kept at 4 °C and carried to ARF, ICDDR’B, using a proper cool-box.

#### Immunization of guinea pigs

Forty-nine guinea pigs (GPs) were divided into two groups, nine in the control group and forty in the experimental group. Forty GPs were further divided into eight groups (A–H), five in each, whereas nine GPs of the control group were divided into three (X, Y and Z) groups, three in each (Table S3). Four experimental groups of GPs were inoculated with four antigen preparations of B1, and the other four groups were immunized for antigen preparation of B3. Then 1.0 ml of each antigen were injected subcutaneously in each GP. The control groups X and Y were inoculated with inactivated monovalent FMDV vaccine types O and A. These vaccines were prepared from the two recently reported vaccine strains BAN/TA/Dh-301/2016 (serotype-O) and BAN/CH/Sa-304/2016 (serotype-A) [31,32]. The control group-Z was inoculated with only 1.0 ml PBS, mixed with an adjuvant. The booster immunization was performed on day 14 with similar antigens in each group. All the animals were sacrificed on day-28, and blood was collected aseptically by direct cardiac puncture. Serum samples were prepared from the blood samples of each animal by centrifugation and were stored until further use.

#### Detection of anti-B1 and anti-B3 antibody in GP serum

The presence of antibodies in the polyclonal GP serum was determined by Western blotting the B1 and B3 antigens. The WB analysis of B1 was performed using both anti-B1 sera and serum of control group-X. Similarly, B3 was blotted by anti-B3 serum and control group-Y serum. Goat anti-guinea pig IgG antibody (Invitrogen, USA) was used as secondary antibody and SuperSignal WestPico PLUS Chemiluminescent Substrate and ChemiDoc Imaging System were used for image development.

#### Virus neutralization tests

To determine each group’s neutralizing antibody titre in serum, virus neutralization tests (VNT) have been performed on monolayers of BHK-21 cells using Karber method [33]. One hundred TCID_50_ of BAN/TA/Dh-301/2016 (serotype-O) strain was used to quantify SN_50_ titre animals immunized with different concentrations of B1 antigen, and BAN/CH/Sa-304/2016 (serotype-A) was used for B3 antigens. The neutralizing efficiency of 100 µg per dose of B1 antigen (group A) was further assayed using serotype-O strains BAN/NA/Ha-156/2013 and BAN/BO/Na-161/2013. In contrast, the efficiency of group-E (100 µg per dose of B3) was assayed using serotype-A strains BAN/DH/Sa-310/2017 and BAN/GA/Sa-197/2013. The neutralizing antibody litres were expressed as the log10 value of the reciprocal of the last dilution that neutralized 100 tissue culture infective dose 50 % (TCID50) of FMDV in 50 % of the wells. All control serum was also tested for determining the SN_50_ titre similarly. To detect the protective titre for FMDV strains, the SN_50_ titre above 1.2 has been considered effective, which was shown in previous reports recognizing the anti-FMDV immune response of guinea pigs as a model for evaluation of vaccine potency in cattle [34,35].

#### Statistical analysis

Statistical analyses of all the raw data of the experiment were performed in R software version 4.0.5 [36,37] and the graphs were built in ggplot2, an open-source R package [38]. We used the one-way ANOVA test to compare the antibody litres raised among the different vaccination doses. When the overall difference across the overall groups was significant, we computed Tukey HSD (Tukey Honest Significant Differences, R function: TukeyHSD) for performing multiple pairwise comparisons between the means of groups. The function TukeyHSD takes the fitted ANOVA as an argument. Analyses were carried out at a 95 % confidence level and *p*-values less than 0.05 were considered statically significant.

## Results

### Designing of chimaeric proteins of FMDV serotype-O

#### *In silico* construction and analysis of multi-epitope recombinant proteins B1 and B3

**Fig. 1.**
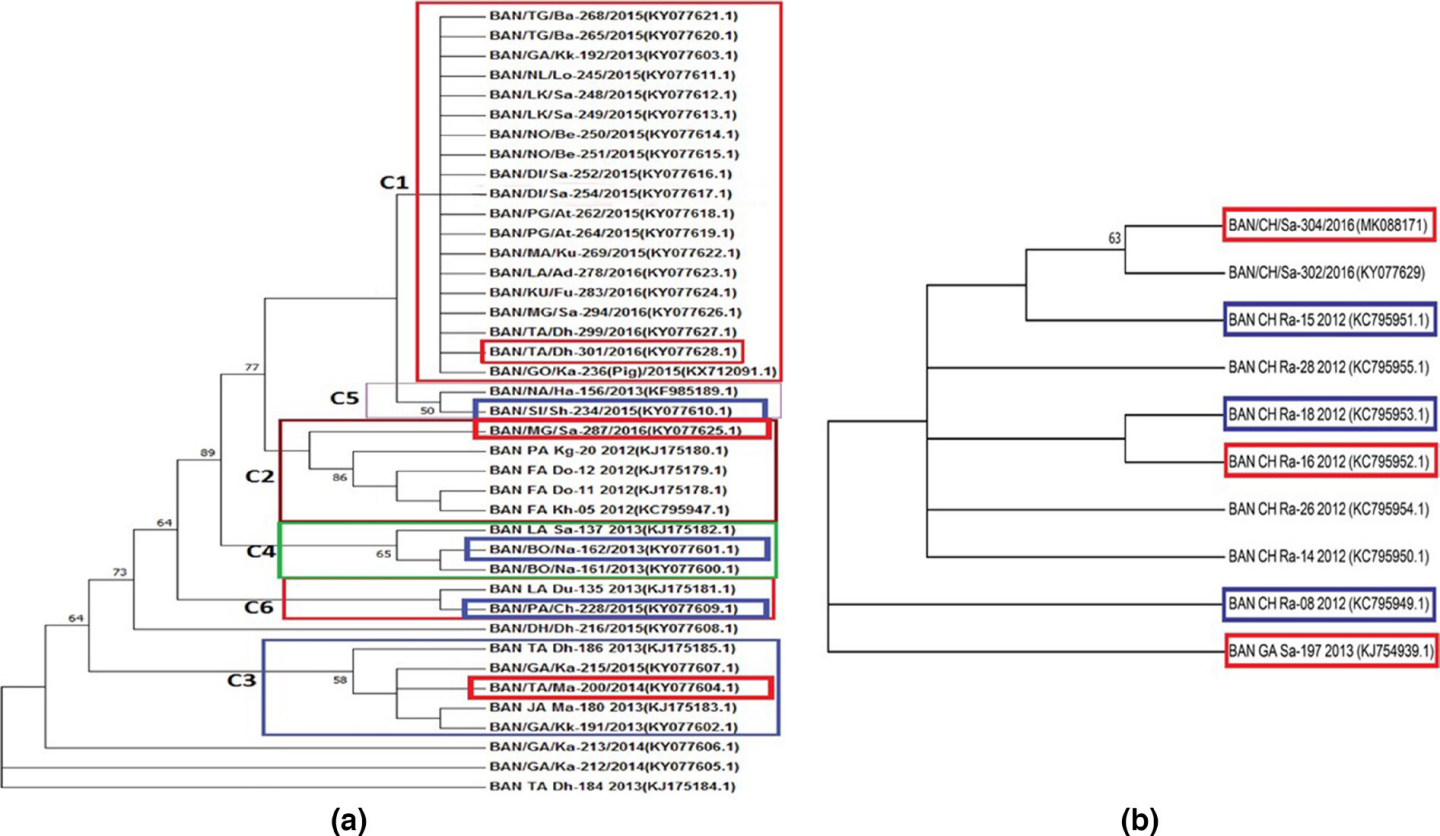
Phylogenetic analyses for variant selection to construct multi-epitope chimaeric proteins of FMDV serotype O and serotype A. (**a**) Phylogenetic analysis of VP1 (121–211 amino acid residues) of 40 Foot-and-Mouth Diseases Virus (FMDV) type O circulating in Bangladesh from 2012 to 2016. The prototype sequences are induced from GenBank. The tree was inferred using neighbour-joining method and simulation with 1000 bootstrap replication value. The tree was condensed with 5 % cut off value. The phylogenetic analysis was directed in mega-7.0. Phylogenetic tree has enabled us to differentiate six clusters. C1 cluster contains 19 sequences, C2 contains five sequences, C3 contains five sequences, C4 contains three sequences, C5 contains two sequences and C6 contains two sequences. There are also four sequences, that did not form any cluster. Representative sequences were selected from phylogenetic tree for recombinant proteins. BAN/TA/Dh-301/2016, BAN/MG/Sa-287/2016 and BAN/TA/Ma-200/2014, lineages have been taken from C1, C2 and C3 clusters respectively (red marking). BAN/BO/Na-162/2013, BAN/SI/Sh-234/2015 and BAN/PA/Ch-228/2015 lineages have been taken from C4, C5 and C6 clusters respectively (blue marking). From clusters C1, C2 and C3, both G-H loop and C-termini have been taken for recombinant proteins. From C4, C5 and C6, only G-H loop has been taken to construct recombinant proteins. (**b**) Phylogenetic tree of serotype A was also constructed in the same way described above and the representative G-H loop and C-terminal (red marked) and only G-H loop (blue marked) were taken for chimaeric proteins reconstruction. (**Bootstrap values less than 50 have not been shown).

**Fig. 2.**
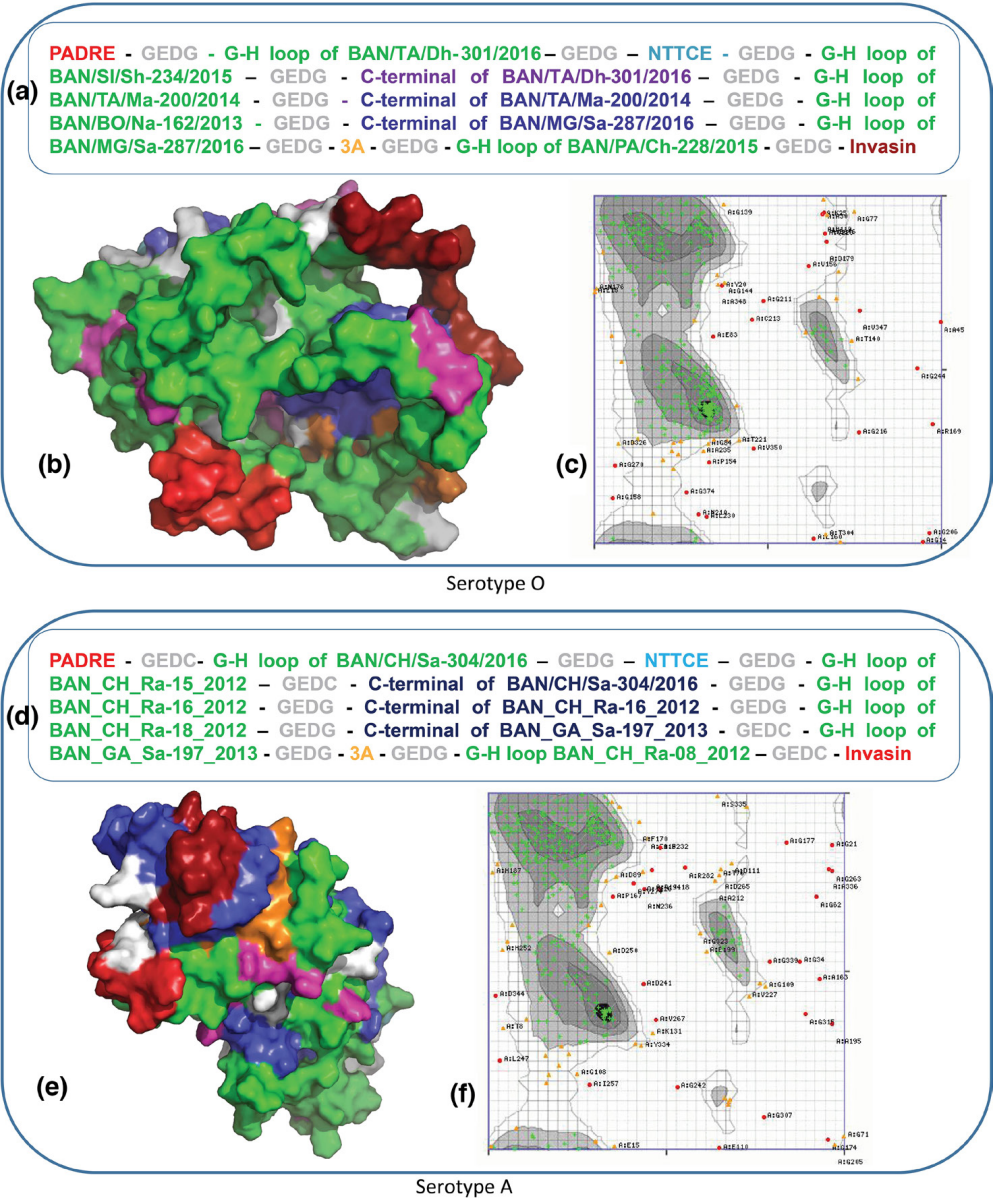
Design, reconstruction and structural validation of multi-epitope chimaeric proteins as a vaccine candidate for serotype A and serotype O. (**a**) Orientation of epitopes with spacer to constract B1 multi-epitope vaccine agaist serotype O; (**b**) tertiary structure of B1 and different colour represent different epitope. Here, red represents PADRE, Invasin; grey represents spacer; green represents G-H loops, blue represents C-terminal epitopes, cyan represents NTTCE, orange represents 3A and finally megenta represents the RGD motifs of G-H loops. (**c**) Ramachandran plot of B1 to structural validation. This plot shows that 84.28 % residues were in highly preferred observaton as green crosses, 9.54 % resdues were in preferred observaton as brown crosses and rest 6.19 % were in questionable observation as red crosses. (**d**) Orientation of epitopes with spacer to constract B3 multi-epitope vaccine agaist serotype A. (**e**) Tertiary structure of B3 and different colour represent different epitope. Here, Red represents PADRE, Invasin; Grey represents spacer; green represents G-H loops, blue represents C-terminal epitopes, cyan represents NTTCE, orange represents 3A and finally megenta represents the RGD motifs of G-H loops. (**f**) Ramachandran plot of B1 to structural validation. This plot shows that 80.58 % residues were in highly preferred observaton as green crosses, 12.60 % resdues were in preferred observaton as brown crosses and rest 6.82 % were in questionable observation as red crosses.

To construct the chimaeric vaccine candidates, B1 and B3, multiple B-cell epitopes and T-cell epitopes were selected from the variable regions of VP1 capsid proteins of FMDV serotype O and A, respectively. For B-cell epitopes, both the GH-loop region (aa 128–164) and C-terminal region (aa 190–211) of VP1 were included in B1 chimaera from BAN/TA/Dh-301/2016, BAN/MG/Sa-287/2016 and BAN/TA/Ma-200/2014, sequences have been taken from C1, C2 and C3 clusters respectively (Fig. 1a). For T-cell epitopes, two epitopes were included, one of which is in the N-terminal region (aa 25–36 for type-O and aa 29–37 for type-A) of VP1 (Fig. 2a), and the other one is in the 3A protein region of FMDV (Tables1  2). The GEDG spacer between each epitope enhanced the acidic and basic amino acid ratio in the final B1 construct, stabilizing the solubility and cytosolic conformation.

In serotype-A specific chimaera B3, a phylogenetic tree was constructed, including G-H loop and C-terminal sequence of VP1. For inclusion in B3 chimaera, the representative G-H loop and C-terminal both (BAN/CH/Sa-304/2016, BAN/CH/Ra-16/2012, BAN/GA/Sa-197/2013) and only G-H loop (BAN/CH/Ra-15/2012, BAN/CH/Ra-18/2012, BAN/CH/Ra-08/2012) epitopes were selected (Fig. 1b). For lack of cysteine in the epitopes of serotype A strains, the GEDC spacer was inserted in four positions in the B3 construct, keeping the rest of the spacers GEDG (Fig. 2d).

The universal T-cell epitopes, PADRE and Invasin, were inserted at the N-terminal and C-terminal positions of both B1 and B3 constructs (Fig. 2a, d). The computational analysis of DNA encoding B1 and B3 revealed that the gene length is 1 170 bp and 1 149 bp, respectively. The predicted physicochemical properties of B1 and B3 have been listed in Table S4. After *in vitro* validation through SDS-PAGE, Western blot and isoelectric focusing gel electrophoresis, the molecular weight and pI of B1 and B3 were determined 41.0 kDa and 7.7, respectively, whereas for B3 these were 39.0 kDa and 8.5, respectively.

For expression of the recombinant proteins in prokaryotic host BL-21(DE3), the codons of the encoding DNA fragments were optimized using the OptimumGene algorithm. The native genes employed rare tandem codons capable of reducing the efficiency and continuity of translation inside the cytoplasm. These were optimized with the inclusion of optimal codons, upgrading the Codon Adaptation Index (CAI), adjusting the GC content, and removing *cis*-acting elements from the transcripts. The codon optimization of B1 and B3 encoding sequences upgraded the CAI index to 0.85 and 0.87, respectively. The average GC content of recombinant DNA fragments of B1 and B3 were adjusted to 58.13 and 60.20 %, respectively. The codon optimization also nullified the unfavourable peaks to prolong the mRNA half-life and disrupt stem-loop structures to enhance the ribosomal binding and stability.

#### Prediction of secondary and tertiary structures of the recombinant proteins

The 'Mfold' server was used to evaluate the minimum free energy for chimaeric mRNA of codon-optimized sequences. The results showed that the minimum free energy of the best predicted structure for the optimized B1 and B3 constructs were −448.80 kcal mol^−1^ and −466.30 kcal mol^−1^. The first nucleotides at 5′ did not have a long stable hairpin or pseudoknot. Therefore, the binding of ribosomes to the translation initiation site and the following translation process can be readily accomplished in the target host. These outcomes were in agreement with data obtained from the 'RNAfold' web server. Several online programmes were used to predict the secondary structures of the chimaeric proteins, and GOR-IV achieved the best result. Results indicated that total residues of B1 were made up of 77 strands (19.74 %), 96 helices (24.62 %) and 217 random coils (55.64 %); whereas B3 contains 161 helices (42.04 %), 47 extended strands (12.27 %) and 175 random coils (45.69 %).

#### Prediction of 3D structure and physicochemical parameters of the recombinant proteins

B1 and B3 were composed of 390 and 383 amino acid residues encoded by 1170 and 1149 nucleotides sequences, respectively. The presence of the RGD motif on the surface of both recombinant proteins was evident when the 3D structures were analysed using PyMOL. The presence of cysteine residues enhanced tertiary structure stability by forming disulphide bridges (Fig. 2b, e). The 3D model of recombinant proteins was analysed by Ramachandran plot (Fig. 2c, f), which showed more than 80 % amino acid residues of both B1 and B3 reside in highly preferred regions. Expasy’s ProtParam classified the optimized chimaeric proteins B1 and B3 as stable (instability index: 18.83 and 19.61, respectively). Other basic physico-chemical parameters of B1 and B3 chimaeric proteins such as molecular weights were 41.3 kDa and 39.3 kDa, respectively (Table S4). The computed half-life for both proteins was greater than 10 h. The *in silico* predicted isoelectric points of B1 and B3 were determined respectively 7.7 and 8.5.

### Expression and analysis of B1 and B3 proteins

#### Expression, purification and characterization of the chimaeric proteins B1 and B3

**Fig. 3.**
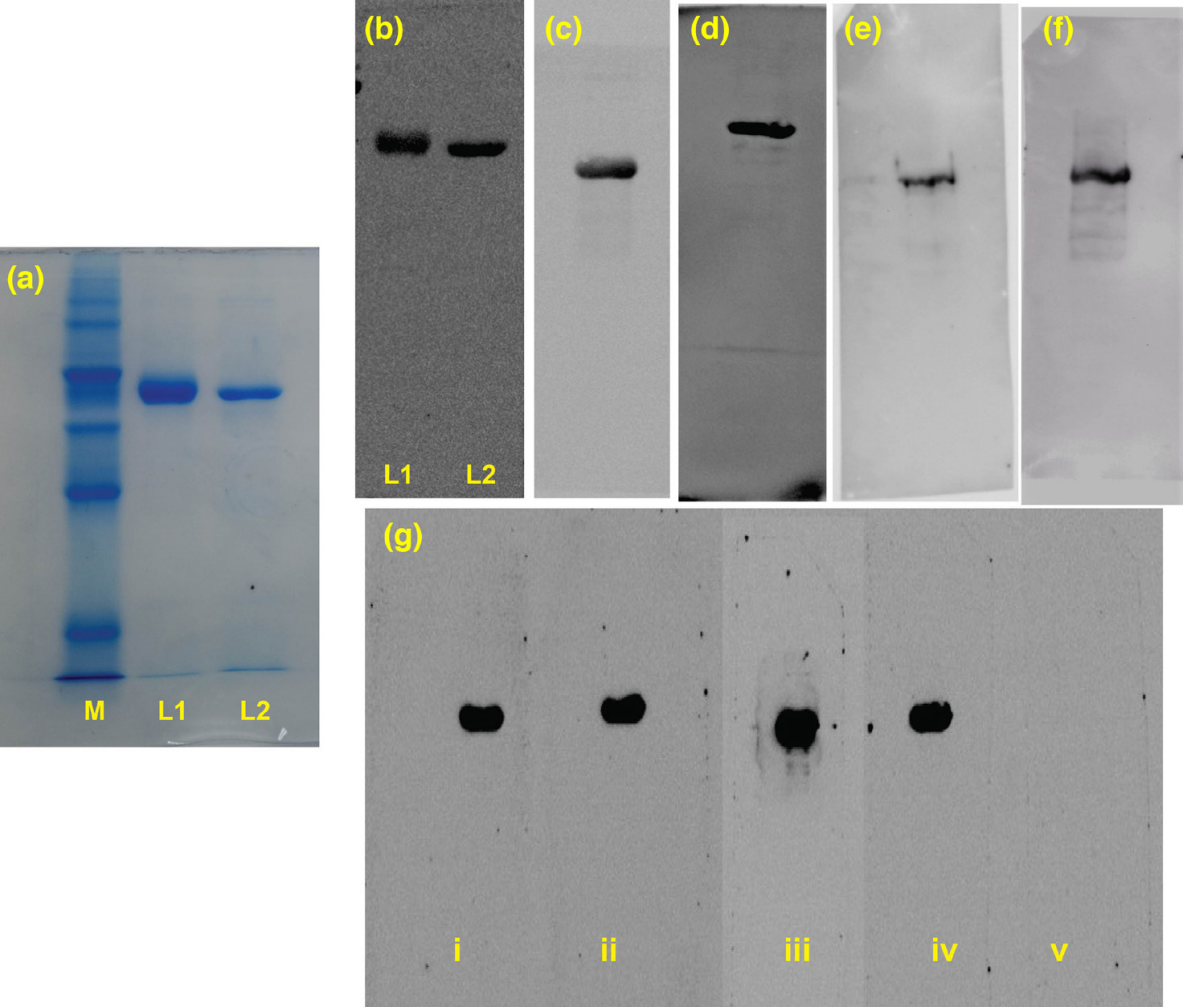
Western blot analysis. (**a**) SDS-PAGE detection of B1 (lane-1) and B3 (lane-2); M=100 kDa proteins marker. (**b**) Detection of 6X-his-tag on B1 (lane-1) and B3 (lane-2) by anti-His-monoclonal antibody. WB detection of (**c**) B1 by monovalent anti-FMDV-O and (**d**) B3 anti-FMDV-A bovine serum. WB detection of (**e**) B1 by monovalent anti-FMDV-O and (**f**) B3 by anti-FMDV-A guinea pig serum. (**g**) WB detection of B1 from GP immunized with (**i**) 100 µg per dose, (ii) 50 µg per dose; B3 detected by GP serum immunized with (iii) 100 µg per dose of B3, (iv) 50 µg per dose of B3; (**v**) negative control serum of GP immunized with PBS only. The monovalent guinea pig and bovine sera were developed by immunization with inactivated BAN/TA/Dh-301/2016 and BAN/CH/Sa-304/2016 vaccine strains, for serotype O and A respectively.

The SDS-PAGE analysis of total cell extract of transformed and induced *E. coli* BL-21(DE3) cells revealed that both B1 and B3 had been expressed in the insoluble fraction of IPTG-induced cells. After purification and desalting, the molecular weight of both the chimaera B1 and B3, confirmed by SDS-PAGE, was determined at 41.0 kDa and 39.0 kDa, respectively (Fig. 3a). The 6X-Histidine tag on the C-terminal of the recombinant proteins was detected by Western blot (Fig. 3b) using an anti-His mouse monoclonal antibody (Thermo Scientific, USA).

The surface antigens of FMDV, incorporated in B1 and B3, have also been detected in the chimaeric proteins through Western blot using monovalent anti-FMDV-O and anti-FMDV-A bovine serum, respectively (Fig. 3c, d, e, f). These WB results indicated that the B-cell epitopes were surface-oriented and readily recognisable by the protective antibodies present in anti-FMDV monovalent sera. Besides, WB of B1 and B3 antigens with homologous anti-B1 and anti-B3 GP sera showed that B1 reacts efficiently with anti-B1 sera from all antigen dose groups (group A-D) and serum of positive control (O-type, group-X) (Fig. 3g). Similarly, B3 also reacted with anti-B3 serum and serum of control group Y (A-type positive control). The serum from the negative control reacted with none of the antigens in WB experiments (Fig. 3g).

#### Serum neutralization efficiency of B1 and B3 developed in the guinea pig models

**Fig. 4.**
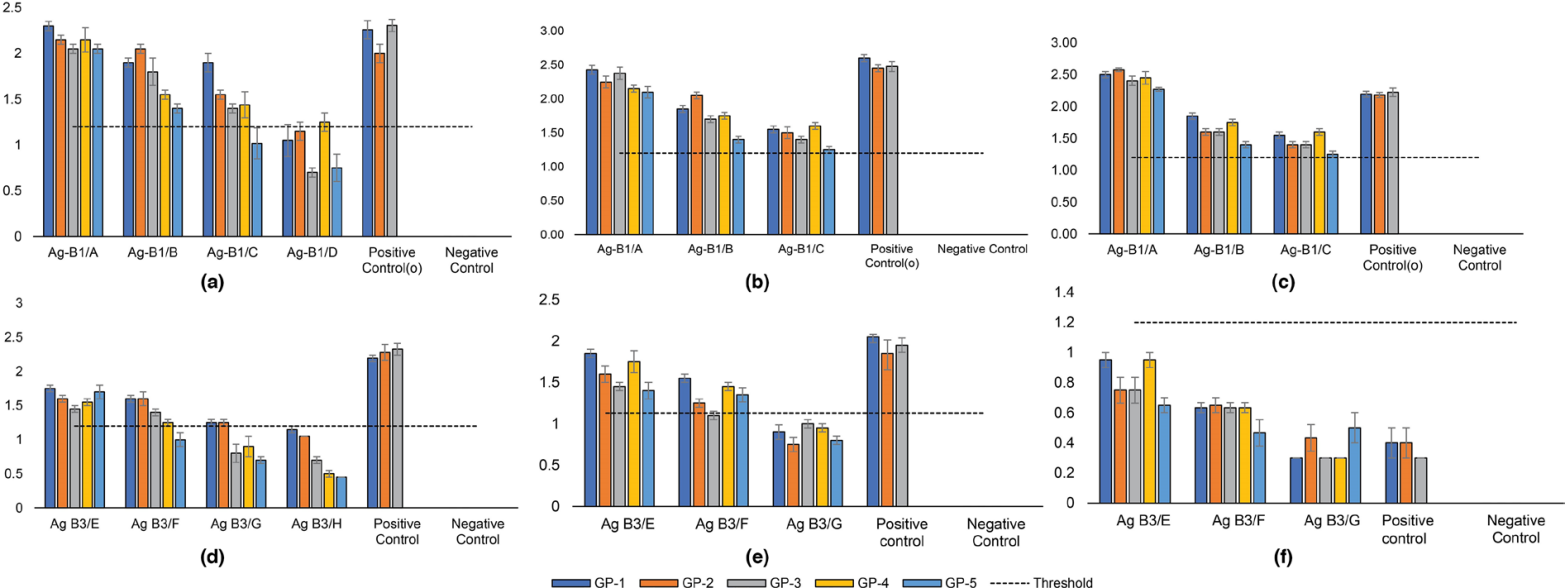
SN50 titre of IgG in anti-B1 serum against (**a**) BAN/TA/Dh-301/2016, (**b**) BAN/NA/Ha-156/2013 and (**c**) BAN/BO/Na-161/2013. SN50 titre of IgG in anti-B3 serum against (**d**) BAN/CH/Sa-304/2016, (**e**) BAN/DH/Sa-310/2017 and (**f**) BAN/GA/Sa-197/2013. The horizontal dashed line indicates the threshold antibody titre 1.2, where the antibody litres≥1.2 (log10) refer the protective level in guinea pig. Error bars represent standard error of the mean (SEM). The X-axis of the bar diagrams indicates GP groups immunized with different concentrations of B1 and B3. The Y-axis represents the SN_50_ titre of serum from each group against specific strain. The lowest Ag-dose (2 µg) provided protective response only in 10–20 % animals, revealed in the VN tests against the recent vaccine strains. These antisera from low-Ag groups were not assessed in subsequent VN tests against other FMDV strains.

The virus neutralization (VN) tests with serum from animals immunized with different doses of B1 showed that the 100 % animals, immunized with both 100 µg (Ag B1/A) and 50 µg (AgB1/B) doses, developed protective humoral response against the most recent vaccine strain BAN/TA/Dh-301/2016. On the other hand, only 80 % (4 out of 5) and 20 % (1 out of 5) of animals developed protective levels of antibody from 10 µg (AgB1/C) and 2 µg (Ag B1/D) doses of B1 antigen (Fig. 4a). Besides, the SN50 titre of animal sera immunized with different doses of B3 antigen showed that animals immunized with 100 µg (Ag B3/E) and 50 µg (AgB3/F) doses of B3 antigen developed protective immune response in 100 and 80% animals of the respective group against the vaccine strain (BAN/CH/Sa-304/2016) for serotype-A (Fig. 4d).

From the VN test with two other circulatory strains of each serotype, it has been revealed that the polyclonal serum from GPs immunized with 100 and 50 µg doses of both antigens, separately, has shown to develop protective immune responses. Specifically, the anti-B1 sera of 100 µg dose showed the development of a protective antibody in 100 % of animals against both BAN/BO/Na-156/2013 and BAN/NA/Ha-161/2013 strains (Fig. 4b, c). In the case of B3 antigen, only strain BAN/DH/Sa-310/2017 has shown to be neutralized in the VN test with anti-B3 sera from both 100 and 50 µg doses of B3 antigens (Fig. 4e). One strain BAN/GA/Sa-197/2013 was neutralized neither by the inactivated monovalent type-A vaccine (positive control) nor by the B3 antigen (Fig. 4f).

**Fig. 5.**
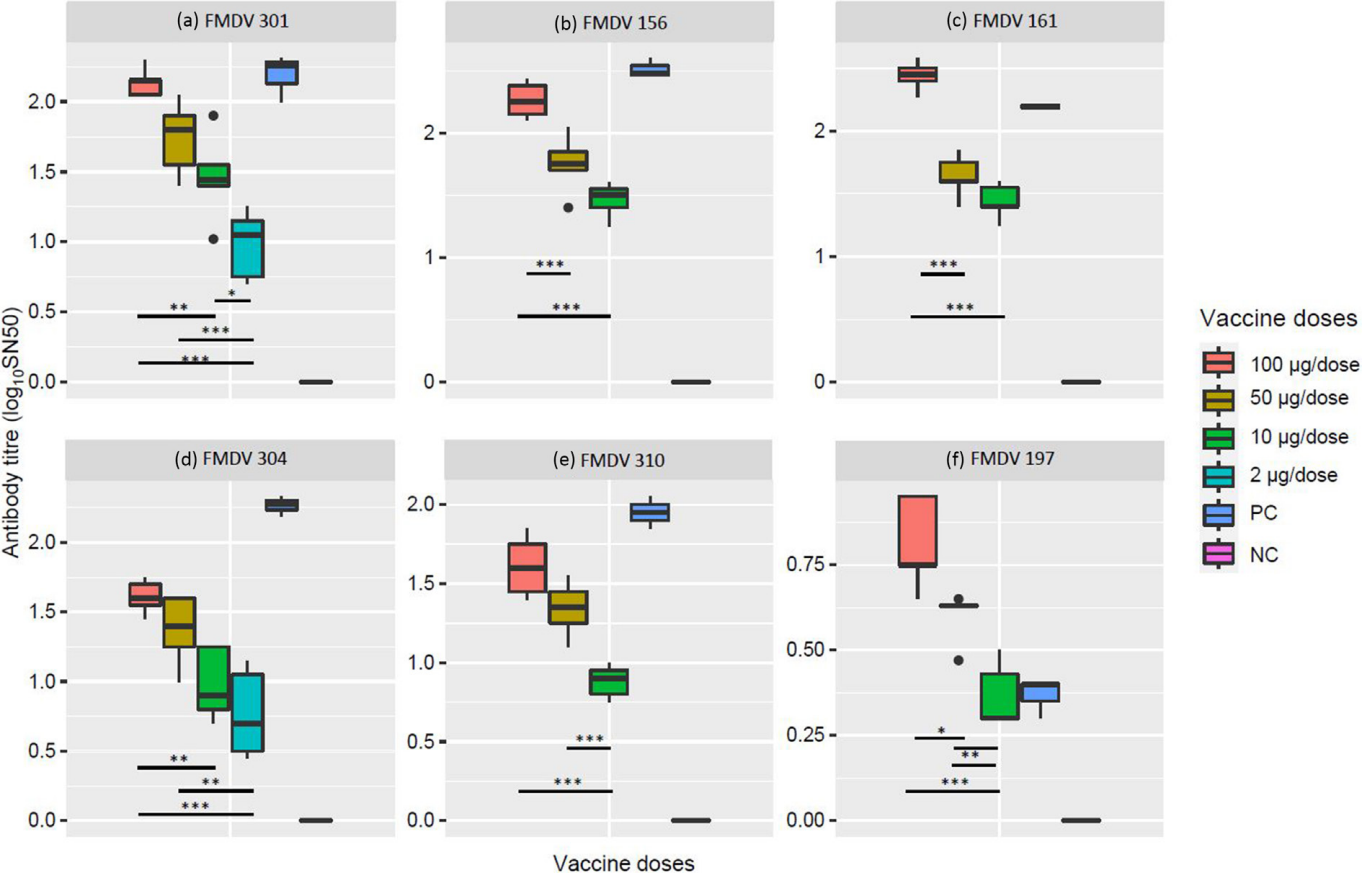
Dose-wise immune response variability of B1 and B3 based vaccines against different field strains of FMDV. SN50 titre of IgG in anti-B1 serum from different doses of B1 against serotype-O strains: (**a**) BAN/TA/Dh-301/2016, (**b**) BAN/NA/Ha-156/2013 and (**c**) BAN/BO/Na*-*161/2013. SN50 titre of IgG in anti-B3 serum multiple doses of B3 against serotype-A strains: (**d**) BAN/CH/Sa-304/2016, (**e**) BAN/DH/Sa-310/2017 and (**f**) BAN/GA/Sa-197/2013. One-way ANOVA test revealed a highly significant (*p*≤0.001) antibody raise between all the vaccinated groups compared and the non-vaccinated (placebo) negative control (NC) (not asterisked in the figure). Statistical significance between other doses has been asterisked at significance codes: 0 ‘***’, 0.001 ‘**’ and 0.01 ‘*’. Adjusted *p* values reported through single-step method. PC=positive control, NC=negative control.

The significance of dose-dependent variation of immune responses against multiple strains of each serotype have been analysed also. One-way ANOVA test revealed a highly significant (*p* ≤0.001) antibody raise among the immunized groups compared to that of non-vaccinated (placebo) negative control (NC). For all of the three FMDV serotype O viruses, there was no statistically significant difference between the positive control (PC) group and the full dose (100 µg/1 ml dose) immunization groups (Fig. 5a). Contrastingly, compared to the chimaeric vaccine (B3), the PC group showed significantly (*P*<0.05) higher antibody level against the serotype A virus strains (BAN/CH/Sa-304/2016 and BAN/DH/Sa-310/2017) (Fig. 5d). Importantly, the chimaeric vaccine (B3) showed significantly (*P*<0.001) higher antibody response than the PC group against FMDV strain BAN/GA/sa-197/2013, that previously reported to be mismatched with the developed inactivated vaccine formulated with BAN/CH/Sa-304/2016 [39,40]. The variance of antibody levels between other groups of vaccination doses has been illustrated in Fig. 5.

## Discussion

Foot-and-Mouth Disease Virus undergoes rapid mutation and transmission to neighbouring countries with animal trade relations, like Bangladesh, India, Myanmar, Nepal etc. Livestock animals are heavily transported across these countries [41,43], which has upgraded the international trade, but also increased the risk of disease transmission [44,46]. For example, transmission of the newly emerged SA-2018 lineage of FMDV serotype-O from India to Bangladesh was recorded in 2021 [47]. Besides, recent studies suggest that the emergence of new lineages and sub-lineages often challenges immune protection provided by the existing vaccine strains in Bangladesh [48]. In-depth genomic analysis showed the presence of ten mutations in the major antigenic sites of the recently published vaccine candidate strain BAN/TA/Dh-301/2016 (MK088170.1), demonstrating significant evolutionary distance from the previously established serotype O vaccine strain, O/India/R2/75 (Accession no. AF204276) [49]. Additionally, a homology analysis of the recently disclosed serotype-A vaccine strain, A/BAN/CH/Sa-304/2016 (MK088171.1) indicated a 91 % resemblance to the vaccine strain, IND40/00 now in use (Accession no. HM854025) [31]. A total of 37 amino acid substitutions, including four in the VP1 capsid protein’s antigenic area, increase the possibility for the circulating strains to evade the immune system, which leads to vaccination failure for the IND40/00 strain. Vaccination failure reports are fairly widespread in our nation and also reported earlier [50]. These investigations stating significant evolutionary distance between the circulating strains and vaccine strains indicates the prime requirement to develop a comprehensive solution for FMD vaccination in Bangladesh.

The development of a subunit vaccine emerged as the most rational and cost-effective method for FMD vaccines [11]. The approach of multi-epitope based chimaeric proteins is considered the best strategy for preventing FMD for their capability of incorporating multiple epitope fragments in a single platform. A heterologous prokaryotic system for recombinant proteins expression has become prevalent and standardized due to the simple cultivation method, low maintenance cost and easy extraction protocols [51]. While using the prokaryotic system for eukaryotic protein expression, one of the significant constraints is codon optimization and the solubility of the expressed proteins [52,54]. Also, the proper three-dimensional configurations of epitope fragments must permit maximum exposure for effective antigen presentation and processing [55]. The artificial recombinant proteins must mimic the spatial arrangement of surface antigens to develop immune responses specific to target pathogens [56]. In this study, these factors have been emphasized in the novel design and orientations of both B1 and B3, respective vaccine candidates for FMDV type O and A prevailing in Bangladesh.

Based on the evolutionary relatedness and cluster formation in the phylogenetic analysis, representative isolates from each of the six clusters (Data S2) for both serotypes, O and A were selected for designing respective chimaeric protein constructs, B1 and B3. This approach was adopted to ensure a broad immune coverage by the chimaeric protein vaccine against multiple circulating variants of FMDV.

Computational analysis of B1 and B3 constructs revealed that insertion of hydrophobic GG spacer sequence results in the formation of two recombinant proteins with much higher isoelectric points, 9.92 and 10.39, respectively. To increase the half-life and stability of tertiary conformation of the artificial proteins, we optimized the isoelectric point of both B1 and B3 by insertion of acidic amino acids in spacer sequences. Each G-H loop fragment of serotype-O strains contained a cysteine residue complementing the probability of disulphide bridge in the final B1 chimaeric proteins. So, GEDG is inserted in twelve spacer positions of B1. For lack of cysteine in epitopes of serotype-A, a GEDC spacer is inserted in four positions of B3 with a GEDG spacer in eight positions.

The overexpressed proteins were abundant in insoluble fractions of total cell extract. The variation of IPTG concentration and cultivation temperature did not alter the solubility pattern of the overexpressed proteins (data not shown). The application of a denaturing agent, 8M urea, did not alter the physicochemical properties, molecular weight, and isoelectric point elucidated in SDS-PAGE gel analysis. So, the orientation and combinations of epitope fragments of B1 and B3 were sustainable throughout the extraction and purification process. The optimized codons of mRNA, disulphide bridges, the higher number of hydrophilic amino acid residues in all twelve spacers sequence (GEDG) and nearly-neutral isoelectric point of B1 have rendered the cytoplasmic stability during heterologous expression and extracellular consistency throughout the purification process. The shortages of disulphide links between the G-H loops and slightly higher isoelectric points have caused partial intracellular degradation of B3, which resulted in a lower yield of B3 than that of B1. Lower yield and cytoplasmic instability due to high pI has also been observed in the expression of other B1-like proteins (B2), with only change in the spacer region (GG). Notably, the isoelectric point of B2 was detected at 9.92 *in silico*. This protein was also expressed but, after purification and SDS-PAGE detection, the proteins were found degraded into smaller fragments indicating cytoplasmic instability, proteolytic degradation and extracellular deterioration in the purification steps.

The characterization and identification of purified B1 and B3 proteins through SDS-PAGE and Western blot analysis revealed the validation of computationally predicted molecular weights and antigenic conformation. The arrangement of multiple epitopes in a single polyprotein must be designed to maintain the antigenicity similar to the infecting pathogen [57,58]. The distortion of antigenic configuration can result in misinterpretation of antigen presentation and the development of non-neutralizing antibodies. The serotype-specific anti-FMDV bovine serum-mediated WB detection of B1 and B3 indicates the presence of serologically active and accessible epitope fragments on the surface of B1 and B3. The three-dimensional surface-orientation of RGD-motifs in G-H loops of the chimaeric proteins have been revealed as capable of proper mimicking of FMDV surface epitopes essential for adaptive immune response. Although both of the chimaeric proteins were purified from insoluble fractions under denaturing conditions, their tertiary configurations were renatured in PBS (pH 7.4) solution which is evident in the subsequent characterization.

The virus neutralization tests revealed that all doses of antigens developed neutralizing antibodies in animal models. However, only higher concentrations, 100 and 50 µg doses of both antigens developed significant titre of neutralizing antibody at the protective level. Specifically, the *in vitro* VN tests of both anti-B1 GP-sera of 100 µg dose and monovalent anti-FMDV-O GP-sera showed the development of similar SN50 titre.

The artificially designed B1 antigen produces a protective level of antibodies against the vaccine strain, BAN/TA/Dh-301/2016 which belong to the recently discovered Ind-2001BD1 sub-lineage from Bangladesh. Besides, the 100 µg dose of B1 also produced a protective level of antibody against BAN/NA/Ha-156/2013 and BAN/BO/Na-161/2013, which belong to the Ind-2001d sub-lineage and recently reported Ind-2001BD2 sub-lineage (Fig. 4b, c). Interestingly, the full dose of B1 antigen (100 µg dose) and the full dose inactivated monovalent FMDV-O vaccine (BAN/TA/Dh/301–2016) produced similar levels of protective response in animal models (Fig. 5a, b, c). These results indicate that the multi-epitope B1 chimaeric proteins possess immunogenic potency against the circulating lineages and recently emerged Ind2001BD1 and Ind2001BD2 sub-lineages of serotype-O strains of FMDV. It can be clearly interpreted that full-dose B1 antigen can develop a protective immune response as efficiently as a full-dose inactivated monovalent type-O vaccine against multiple lineages and sub-lineages of serotype-O. The incorporation of multiple epitopes from evolutionarily diverse subgroups of serotype O have been implemented by the B1 construct design, which was not accomplished in the previous works for protein vaccine development against FMDV serotype-O.

Anti-B3 homologous GP sera from group E (immunized with 100 µg dose) showed that high doses of B3 antigen develop a protective antibody level, but lower than from an inactivated monovalent type-A vaccine. Despite the same antigen load, the lack of proper tertiary orientation and absence of disulphide bridges between epitope fragments have resulted in inefficient antigen processing and ultimately lower neutralizing efficiency of B3. The comparative serum neutralizing efficiency of both artificial B3 proteins and inactivated monovalent type-A FMDV vaccine demonstrated that 100 µg of B3 antigen could not neutralize the infectious virus particles *in vitro* as effectively as the full-dose inactivated vaccine. It is assumed that an increase of B3-antigen concentration shall overcome this limitation and enhance the neutralizing capacity of B3.

The neutralizing efficiency of anti-B3 sera indicated that the phylogenetically related strain BAN/DH/Sa-310/2017 was inhibited by anti-B3 sera in VN tests, except for BAN/GA/Sa-197/2013 serotype-A strain. The amino acid changes within the B-C loop of this strain rendered the serological insufficiency of both recombinant B3 antigen and inactivated vaccine, also reported by Al Amin *et al*. [39,40]. Although, the G-H loop and C-terminal both B-cell epitopes of BAN/GA/Sa-197/2013 were incorporated in the B3 construct, sufficient antibodies were not developed to confer protection. It is noteworthy that the high dose of B3 antigen produced an immune response at a higher level than from the positive control (Fig. 5f), which indicates the optimization of antigen load, inclusion of BC-loop and reconstruction of B3 can enhance expression and immunogenic stability to develop a more potent vaccine candidate for FMDV serotype A in Bangladesh.

To enhance the cell-mediated immune responses, significant T-cell epitopes have been incorporated in both B1 and B3 constructs. These constructs are the very first design containing both universal and FMDV-originated T-cell epitopes in the single platforms along with B-cell epitopes. Although, the cytokine assays for quantitative detection of anti-viral immune response-indicators, like IFN-γ, IL-2, IL-4 could not be accomplished during this study due to poor availability of immunoassay kits for guinea pig cytokine assays during the COVID-19 pandemic. Several reports showed that the inclusion of CpG oligonucleotide enhances the immunogenicity of recombinant proteins-based vaccine candidates [22]. These modifications can be explored to enhance the efficacy of the B1 and B3 antigens in future studies.

Again, the intrusion of the new SA-2018 lineage into the circulation exhibiting most of the mutations in the G-H loop and C-termini of VP1 against circulating as well as vaccine strains was detected in 2021 [47]. Due to this recent emergence and the possibility of future outbreaks, further adjustments in the protein vaccine design are necessary which is easier for the chimaeric protein-based vaccines.

A chimaeric protein-based vaccine would a viable option to address the issue of ensuring immunity against rapidly evolving diverse FMDV genotypes by providing an easy and cost-effective manner of vaccine reconstruction according to the variations in the antigenicity of the virus. Although, many other countries have reported the efficiency of recombinant protein against FMDV, this is the first report from Bangladesh showing the potency of recombinant proteins as vaccine candidates for locally circulating strains of FMDV. Importantly, the robust expression of B1 in prokaryotic cells has provided the exclusive potency as a suitable vaccine candidate for high yield, post-translational stability, neutralization efficacy against multiple lineage and sub-lineages of FMDV serotype-O in Bangladesh. This single vaccine candidate can be further adapted to develop immunity against newly emerged lineages like SA-2018.

This study provides a sustainable strategy for developing recombinant protein vaccines for FMDV in Bangladesh, after in-depth assessment in appropriate field animals through standard procedures. However, the protein-based vaccine can also be provided combinedly with inactivated vaccines, to increase the immune responses against old and newly-emerged strains of FMDV lineages and sub-lineages in Bangladesh and neighbouring countries.

## Conclusion

To control and restrict the transboundary transmission and prevalence of FMD, caused by newly emerging lineages and sub-lineages of FMDV in Bangladesh and neighbouring territories, the application of multiple epitopes based recombinant vaccines will enhance immune diversity targeting most prevalent genotypes. This study presents the very first report on novel designs for FMDV vaccine candidates comprised of multiple epitopes from the most recent sub-lineage. The sero-protection efficiencies of the recombinant proteins should be further assessed through live virus challenge tests in guinea pig and cattle models, along with homologous and heterologous virus strains isolated from different FMD strains endemic in this South Asian region.

## supplementary material

10.1099/acmi.0.000713.v4 Uncited Supplementary Material 1.

## Abbreviations

ARF: animal resource facility
CAI: Codon Adaptation Index
FMD: foot-and-mouth disease
FMDV: foot-and-mouth disease virus
GPs: guinea pigs
ICDDR’B: International Centre for Diarrheal Disease Research, Bangladesh
MGBL: microbial genetics and bioinformatics lab
NC: negative control
NSPs: non-structural proteins
PC: positive control
PCP-FMD: progressive control pathway for foot-and-mouth disease
SAT: Southern African Territories
