# Supplementary material for: Development of recombinant proteins for vaccine candidates against serotypes O and A of Foot-and-Mouth Disease virus in Bangladesh
Source: Access Microbiol. 2024 Jun 24;6(6):000713.v4. doi: 10.1099/acmi.0.000713.v4 (PMC11261717; doi:10.1099/acmi.0.000713.v4)
Supplement: Uncited Supplementary Material 1. [file acmi-6-00713-s001.pdf]

1. Supplementary Tables

Table 1: NCBI accession no. of FMDV Serotype O Isolates used in Phylogenetic analysis

Name of Isolate	Accession No.	Name of Isolate	Accession No.
BAN/TG/Ba-268/2015	KY077621.1	BAN/LA/Ad-278/2016	KY077623.1
BAN/TG/Ba-265/2015	KY077620.1	BAN/KU/Fu-283/2016	KY077624.1
BAN/GA/Kk-192/2013	KY077603.1	BAN/MG/Sa-294/2016	KY077626.1
BAN/NL/Lo-245/2015	KY077611.1	BAN/TA/Dh-299/2016	KY077627.1
BAN/LK/Sa-248/2015	KY077612.1	BAN/TA/Dh-301/2016	KY077628.1
BAN/LK/Sa-249/2015	KY077613.1	BAN/GO/Ka-236(Pig)/2015	KX712091.1
BAN/NO/Be-250/2015	KY077614.1	BAN/NA/Ha-156/2013	KF985189.1
BAN/NO/Be-251/2015	KY077615.1	BAN/MG/Sa-287/2016	KY077625.1
BAN/DI/Sa-252/2015	KY077616.1	BAN PA Kg-20 2012	KJ175180.1
BAN/DI/Sa-254/2015	KY077617.1	BAN FA Do-12 2012	KJ175179.1
BAN/PG/At-262/2015	KY077618.1	BAN LA Sa-137 2013	KJ175182.1
BAN/PG/At-264/2015	KY077619.1	BAN/BO/Na-162/2013	KY077601.1
BAN/MA/Ku-269/2015	KY077622.1	BAN/BO/Na-161/2013	KY077600.1
BAN LA Du-135 2013	KJ175181.1	BAN JA Ma-180 2013	KJ175183.1
BAN/PA/Ch-228/2015	KY077609.1	BAN/GA/Kk-191/2013	KY077602.1
BAN/DH/Dh-216/2015	KY077608.1	BAN/GA/Ka-213/2014	KY077606.1
BAN TA Dh-186 2013	KJ175185.1	BAN/GA/Ka-212/2014	KY077605.1
BAN/GA/Ka-215/2015	KY077607.1	BAN TA Dh-184 2013	KJ175184.1
BAN/TA/Ma-200/2014	KY077604.1	BAN/SI/Sh-234/2015	KY077610.1
BAN FA Do-11 2012	KJ175178.1	BAN FA Kh-05 2012	KC795947.1

Table 2: NCBI accession no. of FMDV Serotype A Isolates used in Phylogenetic analysis

Name of Isolate	Accession No.	Name of Isolate	Accession No.
BAN/CH/Sa-304/2016	MK088171	BAN_CH_Ra-08_2012	KC795949. 1
BAN_CH_Ra-15_2012	KC795951.1	BAN/CH/Sa-302/2016	KY077629
BAN_CH_Ra-16_2012	KC795952.1	BAN CH Ra-28 2012	KC795955.1
BAN_CH_Ra-18_2012	KC795953.1	BAN CH Ra-26 2012	KC795954.1
BAN_GA_Sa-197_2013	KJ754939.1	BAN CH Ra-14 2012	KC795950.1

Supplementary Table-3: Immunization Plan of Experimental guinea pigs

**Animal Group**		**Antigen Type/Conc.**	**No of GP**		**Total No. of Animals**
			B1	B3	
**Experimental Group (n=24; B1= 20; B3=20)**		100 µg/dose	(A) 5	(E) 5	10
		50 µg/dose	(B) 5	(F) 5	10
		10 µg/dose	(C) 5	(G) 5	10
		2 µg/dose	(D) 5	(H) 5	10
**Control Group (n=9)**	Positive Control	Inactivated Type-O monovalent vaccine (BAN/DH/Dh-301/2016)	(X) 3		3
		Inactivated Type-A monovalent vaccine (BAN/CH/Sa-304/2016)	(Y) 3		3
	Negative Control	PBS with Montanide 201Vg	(Z) 3		3
**Total No of Guinea Pig models**<br>• Body Weight: 300-450 gm<br>• Immunization Route: Subcutaneous<br>• Immunization Schedule:<br>Day 1 --- Primary immunization of all GP<br>Day 14– Secondary (Booster) Immunization<br>Day 28 – Blood collection of all groups					49

Supplementary Table-4: Physicochemical parameters of B1 and B3

Characteristics	B1	B3
MW (kDa)	41.3	39.8
Isoelectric Point (pI)	7.75	8.51
No. of Amino Acid	390	383
Recombinant protein solubility Prediction	100.0 percent	100.0 percent
Estimated half-life	>10 hours (*E. coli*, in vivo)	>10 hours (*E. coli*, in vivo)

Supplementary Table-5: SN50 titer against FMDV strains of different groups vaccinated by different doses of B1 and B3

Animal group		GP-1	GP-2	GP-3	GP-4	GP-5	Animal group		GP-1	GP-2	GP-3	GP-4	GP-5
**Ag-B1/A**	BAN/TA/Dh-301/2016	2.3	2.15	2.05	2.15	2.05	**Ag-B3/E**	BAN/CH/Sa-304/2016	1.75	1.6	1.45	1.55	1.7
	BAN/NA/Ha-156/2013	2.43	2.25	2.38	2.15	2.10		BAN/DH/Sa-310/2017	1.75	1.6	1.45	1.55	1.7
	BAN/BO/Na-161/2013	2.50	2.58	2.40	2.45	2.27		BAN/GA/Sa-197/2013	0.95	0.75	0.75	0.95	0.65
**Ag-B1/B**	BAN/TA/Dh-301/2016	1.9	2.05	1.8	1.55	1.4	**Ag-B3/F**	BAN/CH/Sa-304/2016	1.6	1.6	1.4	1.25	1
	BAN/NA/Ha-156/2013	1.85	2.05	1.7	1.75	1.4		BAN/DH/Sa-310/2017	1.55	1.25	1.1	1.45	1.35
	BAN/BO/Na-161/2013	1.85	1.6	1.6	1.75	1.4		BAN/GA/Sa-197/2013	0.63	0.65	0.63	0.63	0.47
**Ag-B1/C**	BAN/TA/Dh-301/2016	1.90	1.55	1.40	1.44	1.02	**Ag-B3/G**	BAN/CH/Sa-304/2016	1.25	1.25	0.8	0.9	0.7
	BAN/NA/Ha-156/2013	1.55	1.50	1.40	1.60	1.25		BAN/DH/Sa-310/2017	0.90	0.75	1.00	0.95	0.80
	BAN/BO/Na-161/2013	1.55	1.40	1.40	1.60	1.25		BAN/GA/Sa-197/2013	0.30	0.43	0.30	0.30	0.50
**Positive Control (O)**	BAN/TA/Dh-301/2016	2.26	2.00	2.31			**Positive Control (A)**	BAN/CH/Sa-304/2016	2.19	2.28	2.33		
	BAN/NA/Ha-156/2013	2.60	2.45	2.48				BAN/DH/Sa-310/2017	2.05	1.85	1.95		
	BAN/BO/Na-161/2013	2.19	2.18	2.22				BAN/GA/Sa-197/2013	0.40	0.40	0.30		
**Negative Control**		0	0	0			**Negative Control**		0	0	0		

2. Supplementary Figures

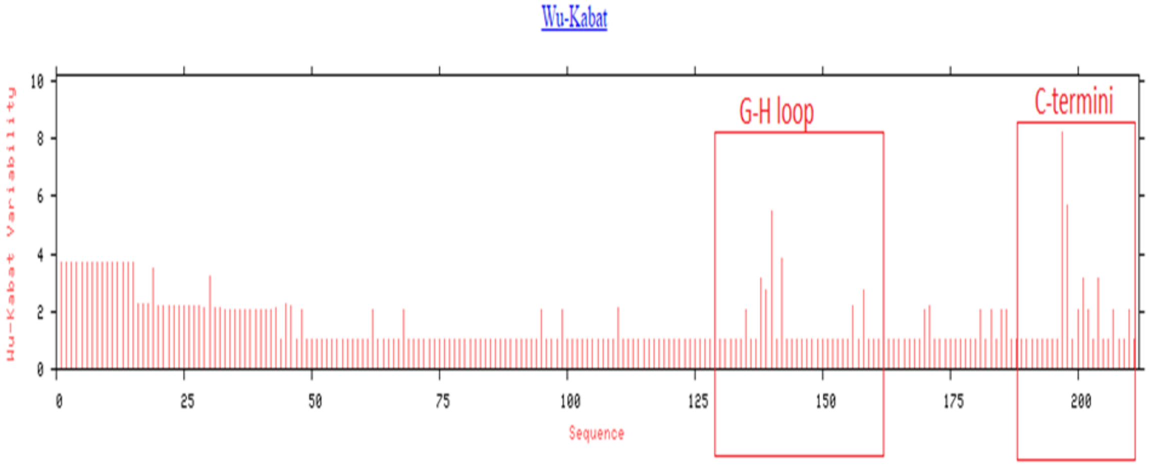

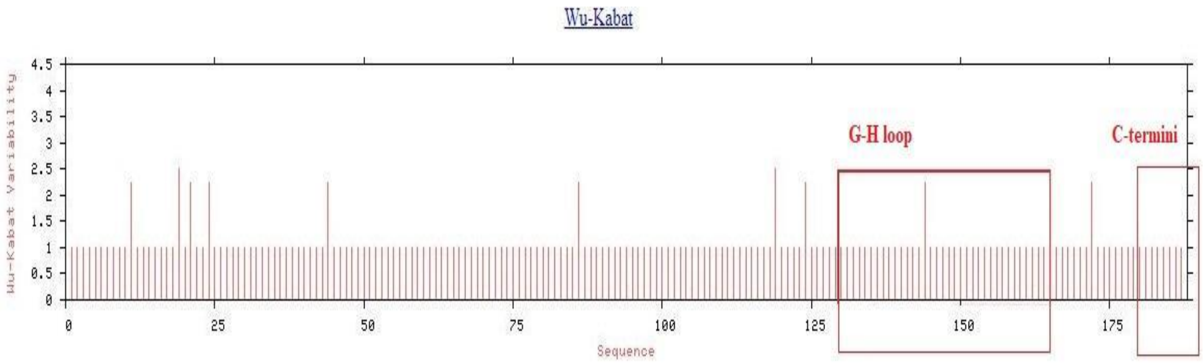

Suppl. Figure 1: Wu-Kabat protein variability plot for VP1 type O and A circulating in Bangladesh. Variability was most observed at G-H loop [135-158] and C-termini [195-211] positions. (a) Variability plot of the whole VP1 protein of serotype O, (b) Variability plot of the whole VP1 protein of serotype A.

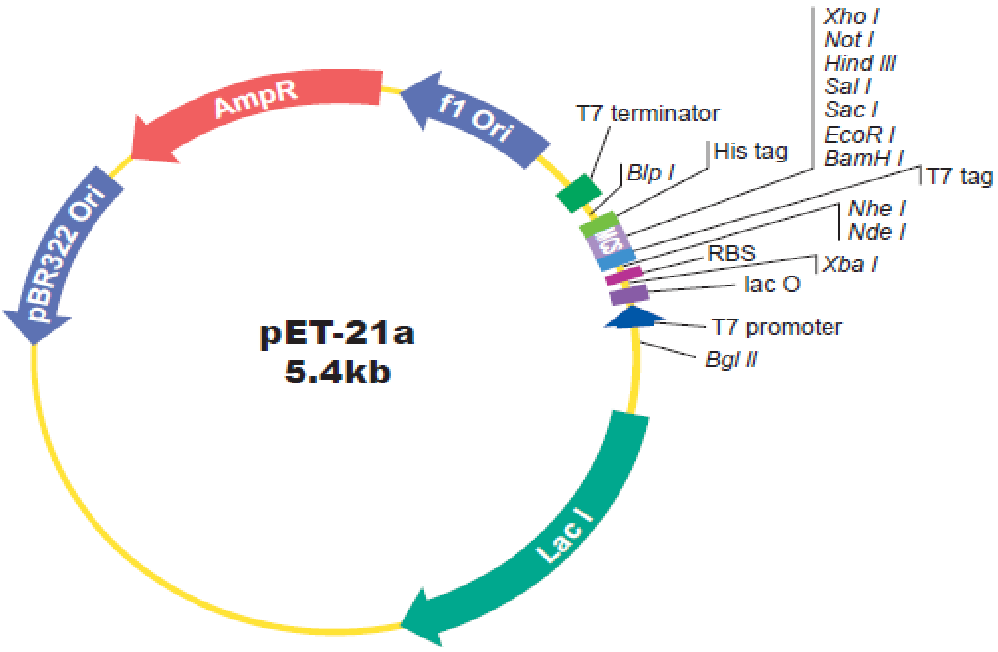

Suppl. Figure-2: Map of pET 21a(+) expression vector with selectable marker

Insertion sequences between epitope fragments of B1 and B3

Insert position	B1	B3
1	GEDG	GEDC
2	GEDG	GEDG
3	GEDG	GEDG
4	GEDG	GEDC
5	GEDG	GEDG
6	GEDG	GEDG
7	GEDG	GEDG
8	GEDG	GEDG
9	GEDG	GEDC
10	GEDG	GEDG
11	GEDG	GEDG
12	GEDG	GEDC

B1

AKFVAAWTLKAAA GEDGTVYNGNCKYGEGAVTNVRGDLQVLAQKATRTL
PTS FNGEDGVTPQNQINVLGEDGTVYNGNCKYGEGAVTNVRGDLQVLAQ
KATRTLPTS FNGEDGPLLAIHPEQARHKQKIVAPVKQ GEDGTVYNGNCKY
GES NVPNVRGDLQVLAQKAARPLPTS FN GEDGPLLAIHPS EARHKQKIVAP
VKQGEDGTVYNGNCKYGEGAVTNVRGDLQVLAQKAARTLPTS FN GEDGP
LLAIHPGQARHKQKIVAPVKQ GEDGTVYNGNCKYGEGAVTNVRGDLQVL
AQKAARTLPTS FNGEDGAAIEFFEGMVHDS IK GEDGTVYNGNCKYGGS DV
ANVRGDLQVLAQKAARPLPTS FN GEDGTAKS KKFPS YTATYQF

B3

AKFVAAWTLKAAA GEDCVYNGTNKYS AAS GRARGDLGQLAARVAAQLPA
S FNFGGEDGVKIGNVS PTGEDGVYNGTNKYS AAS GRVRGDLGQLAARVAA
QLPAS FNFGGEDCAVEVS S QDRHKQKIIAPAKQGEDGVYNGTNKYS AAS GR
VRGDLGQLAARVAAQLPAS FNFG GEDGAVEVS S QDRHKQKIIAPAKQGED
GVYNGTNKYS AAS GRVRGDLEQLAARVAAQLPAS FNFG GEDGAVEVLS QD
RHKQKIIAPAKQ GEDCVYNGTNKYS AAS GRVRGDLGQLAARVAAQLPAS F
NFGGEDGAAIEFFEGMVHDS IK GEDGVYNGTNKYS AAS GRVRGDLGQLAA
RVAAQLPAS FNFGGEDCTAKS KKFPS YTATYQF

Suppl. Figure-3: Insertion sequences and amino acid sequences of B1 and B3.
